# The Potential for CH_4_ Production by Syntrophic Microbial Communities in Diverse Deep Aquifers Associated with an Accretionary Prism and its Overlying Sedimentary Layers

**DOI:** 10.1264/jsme2.ME19103

**Published:** 2020-01-11

**Authors:** Makoto Matsushita, Shugo Ishikawa, Kenta Magara, Yu Sato, Hiroyuki Kimura

**Affiliations:** 1 Department of Geosciences, Faculty of Science, Shizuoka University, Shizuoka, Shizuoka 422–8529, Japan; 2 Institute for Geo-Resources and Environment, National Institute of Advanced Industrial Science and Technology (AIST), Tsukuba, Ibaraki 305–8566, Japan; 3 Department of Biotechnology, Graduate School of Engineering, Osaka University, Suita, Osaka 565–0871, Japan; 4 Research Institute of Green Science and Technology, Shizuoka University, Shizuoka, Shizuoka 422–8529, Japan

**Keywords:** accretionary prism, deep aquifer, methane production, methanogenic archaea, fermentative bacteria

## Abstract

Accretionary prisms are thick masses of sedimentary material scraped from the oceanic crust and piled up at convergent plate boundaries found across large regions of the world. Large amounts of anoxic groundwater and natural gas, mainly methane (CH_4_), are contained in deep aquifers associated with these accretionary prisms. To identify the subsurface environments and potential for CH_4_ production by the microbial communities in deep aquifers, we performed chemical and microbiological assays on groundwater and natural gas derived from deep aquifers associated with an accretionary prism and its overlying sedimentary layers. Physicochemical analyses of groundwater and natural gas suggested wide variations in the features of the six deep aquifers tested. On the other hand, a stable carbon isotope analysis of dissolved inorganic carbon in the groundwater and CH_4_ in the natural gas showed that the deep aquifers contained CH_4_ of biogenic or mixed biogenic and thermogenic origins. Live/dead staining of microbial cells contained in the groundwater revealed that the cell density of live microbial cells was in the order of 10^4^ to 10^6^‍ ‍cells‍ ‍mL^–1^, and cell viability ranged between 7.5 and 38.9%. A DNA analysis and anoxic culture of microorganisms in the groundwater suggested a high potential for CH_4_ production by a syntrophic consortium of hydrogen (H_2_)-producing fermentative bacteria and H_2_-utilizing methanogenic archaea. These results suggest that the biodegradation of organic matter in ancient sediments contributes to CH_4_ production in the deep aquifers associated with this accretionary prism as well as its overlying sedimentary layers.

Accretionary prisms are thick layers of sedimentary material piled up at convergent plate boundaries. This material, which was originally deposited on a subducting ocean plate, is accreted onto a non-subducting continental plate during the subduction process (Taira *et al.*, 1982). Accretionary prisms are found across large regions of the world, including Alaska and Washington in the United States, New Zealand, Chile, Peru, Indonesia, Taiwan, and Japan (Kano *et al.*, 1991; Reed *et al.*, 2002; Hervé *et al.*, 2013; Lee *et al.*, 2017).

The Shimanto Belt, an accretionary prism found in southwest Japan, was mainly formed during the Cretaceous and Paleogene periods and is traceable for 1,800‍ ‍km in parallel with the Nankai Trough and Ryukyu Trench (Kano *et al.*, 1991; Taira *et al.*, 1992). The Shimanto Belt originated from ancient marine sediment deposited on the Philippine Sea Plate (Kano *et al.*, 1991). This sediment is approximately 10‍ ‍km thick and contains layers of water-bearing permeable sandstone and water-impermeable shale that are rich in complex organic matter (Maki *et al.*, 1980; Taira *et al.*, 1982). Rainwater and seawater recharge through faults or fracture zones formed by earthquakes that occur at the plate boundaries, and these waters then collect in the deep aquifers of accretionary prisms in which they become anoxic over time (Sakata *et al.*, 2012; Baito *et al.*, 2015). Deep aquifers contain large amounts of groundwater with dissolved and free phase natural gas, mainly methane (CH_4_) (Kimura *et al.*, 2010; Matsushita *et al.*, 2016; Matsushita *et al.*, 2018).

The origin of CH_4_ in natural gas reserves in subsurface sedimentary deposits is biogenic (formed by methanogenic archaea) or thermogenic (formed by the thermal degradation of organic matter in sedimentary layers). Previous studies investigated CH_4_ production processes in the Shimanto Belt’s deep aquifers, which are affected by rainwater and seawater flowing down from surface environments, and reported that the syntrophic biodegradation of organic matter by hydrogen (H_2_)-producing fermentative bacteria and H_2_-utilizing methanogens, as well as a thermogenic reaction, contributes to CH_4_ production in these deep aquifers (Kimura *et al.*, 2010; Matsushita *et al.*, 2016; Imachi, 2017; Matsushita *et al.*, 2018; Tamaki, 2019). On the other hand, Matsushita *et al.* (2016; 2018) demonstrated that groundwater in the deep aquifers associated with the Shimanto Belt had different chemical characteristics from site to site, based on measurements of temperature, salinity, chemical components, and oxygen and hydrogen stable isotope ratios. These findings suggest that the subsurface environments of the accretionary prisms are not constant and are affected by the geological and geochemical features of each region.

The anaerobic deep aquifers associated with accretionary prisms contain large amounts of CH_4_ (Kimura *et al.*, 2010; Sakata *et al.*, 2012). CH_4_ is a potential greenhouse gas and important energy resource. However, all of the research on CH_4_ production processes conducted to date has targeted deep aquifers that are affected by rainwater and seawater derived from surface environments. Accretionary prisms and their overlying sedimentary layers that contain deep aquifers are influenced by magmatic CO_2_ derived from the deep subsurface environments of active volcanoes or by ancient seawater containing high concentrations of iodine and bromine (Kato, 1985). Mayumi *et al.* (2013) previously reported that a high concentration of CO_2_ may change microbial CH_4_ production pathways in subsurface oil reservoirs. Additionally, iodine and bromine are generally known to exhibit antimicrobial activity (Odlaug, 1981). These factors may affect the microbial activity, community structure, and CH_4_ production pathway in deep aquifers associated with accretionary prisms; however, there is currently no experimental evidence to support this.

We herein investigated the deep aquifers associated with the accretionary prism and its overlying sedimentary layers in the southeastern part of Kyushu Island, Japan, near active volcanoes. To characterize each deep aquifer, we analyzed groundwater and natural gas samples with chemical and isotopic techniques. The aim of the present study was to clarify whether CH_4_ production by syntrophic communities of H_2_-producing fermentative bacteria and H_2_-utilizing methanogens occur universally in deep aquifers associated with accretionary prisms. Therefore, we investigated microbial community structures and their potential for CH_4_ production using microbial cell counts, a 16S rRNA gene analysis, and anaerobic culture methods. An understanding of the underlying mechanisms and potential for CH_4_ production in deep aquifers associated with accretionary prisms, which are distributed worldwide and at which geodynamic phenomena, such as huge earthquakes and fault formation, occur, may provide novel insights into greenhouse gas emissions and global warming.

## Materials and Methods

### Study sites

**Fig. 1.**
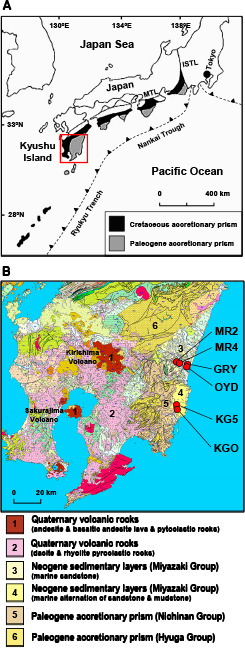
(A) The location of the accretionary prism in southwest Japan, known as the Shimanto Belt, was taken from Kano *et al.* (1991). The red square in map A is enlarged in the geological map in panel B. MTL, Median Tectonic Line; ISTL, Itoigawa-Shizuoka Tectonic Line. (B) The geological map modified from a 1:200,000 seamless digital geological map of Japan that was published by the Geological Survey of Japan, AIST (2014). The red circles indicate the location of the deep wells used for sampling.

The present study was conducted in the southeastern part of Kyushu Island, Japan, near some of Japan’s most active volcanoes (*e.g.*, Kirishima Volcano and Sakurajima Volcano). The accretionary prism known as the Nichinan Group (mainly Paleogene) in the Shimanto Belt is distributed across this region (Fig. 1). The Nichinan Group is composed principally of sandstone, siltstone, conglomerate, and basalt (Kato, 1985). Sedimentary layers that unconformably overlay the Nichinan Group are referred to as the Miyazaki Group (mainly Neogene). The Miyazaki Group is mainly composed of sandstone, mudstone, and conglomerate that were deposited in a forearc basin (Suzuki, 1987; Oda *et al.*, 2011).

### Sampling of groundwater and natural gas

Anoxic groundwater and natural gas samples were collected from deep aquifers associated with the Nichinan and Miyazaki Groups through six deep wells: GRY, OYD, MR2, MR4, KGO, and KG5 (Fig. 1B and Table S1). These wells were drilled down to 810 to 1,301 m and constructed from tight steel-casing pipes including strainers (Table 1) (Kato *et al.*, 2011; Sakata *et al.*, 2012). Groundwater at these wells is anoxically drawn up to ground level by a water pump. In the present study, to prevent contamination by air and water remaining inside the pipe, 200 to 700 tons of groundwater was pumped before sampling. Groundwater samples were collected under anoxic conditions into autoclaved serum bottles and polycarbonate bottles using a sterile silicone tube. The concentrations of dissolved natural gas were so high that gas exsolved at ground level. Natural gas samples were collected into autoclaved 100-mL serum bottles underwater. Serum bottles were tightly sealed with sterile butyl-rubber stoppers and aluminum crimps to prevent contamination by air. Collected samples were transferred to the laboratory on ice and then stored at 4°C until later use.

### Physicochemical and stable isotope analyses

The temperature, pH, oxidation-reduction potential (ORP), and electrical conductivity (EC) of groundwater samples were measured at the outflows of the wells. Temperature was measured with a CT-460WR thermometer (Custom). pH was measured with an HM-20P portable meter (DKK-TOA). ORP and EC were measured with RM-20P and CM-21P portable meters (DKK-TOA), respectively. The concentrations of cations (Na^+^, Ca^2+^, Mg^2+^, K^+^, and NH_4_^+^) and anions (CI^–^, Br^–^, I^–^, F^–^, PO_4_^3–^, NO_3_^–^, SO_4_^2–^, acetate, and formate) in groundwater were analyzed using an ICS-1500 ion chromatography system (Dionex). The concentration of HCO_3_^–^ was measured by the separation titration of groundwater with H_2_SO_4_ using the digital titrator model 16900 (Hach) according to the manufacturer’s instructions. Sulfide was analyzed using a No. 211 sulfide ion detector tube (Gastec). Dissolved organic carbon (DOC) in the groundwater, filtered through a pre-combusted Whatman GF/F glass microfiber filter (GE Healthcare), was measured on a TOC-V total organic carbon analyzer (Shimadzu).

H_2_, N_2_, O_2_, CO_2_, and CH_4_ concentrations in natural gas were measured with a GC-2014 gas chromatograph (GC) (Shimadzu) equipped with a thermal conductivity detector (TCD) and packed column (ShinCarbon ST, 6.0 m×3.0 mm i.d.; Shinwa Chemical Industries). The GC conditions used were as follows: injector temperature, 170°C; column oven temperature, 150°C; detector temperature, 170°C. Argon was used as a carrier gas at a constant flow mode of 50 mL min^–1^. CH_4_, C_2_H_6_, and C_3_H_8_ concentrations were measured with a GC-2014 GC equipped with a flame ionization detector and packed column (Sunpak-A, 2.0 m×3.0 mm i.d.; Shinwa Chemical Industries). The GC conditions used were as follows: injector temperature, 100°C; column oven temperature, 65°C; detector temperature, 100°C. N_2_ was used as a carrier gas at a constant flow rate of 50 mL min^–1^. Samples were analyzed in triplicate. The confidence limits of the measurement were 0.01‍ ‍vol.% for H_2_, N_2_, O_2_, CO_2_, and CH_4_, and 0.001‍ ‍vol.% for C_2_H_6_ and C_3_H_8_.

The stable hydrogen and oxygen isotope ratios of groundwater samples (D/H and ^18^O/^16^O) were measured with a DLT-100 liquid water isotope analyzer (Los Gatos Research) following the procedures described by Dawson *et al.* (2015). The stable carbon isotope ratio (^13^C/^12^C) of dissolved inorganic carbon (DIC; consisting mainly of HCO_3_^–^) was measured with a Trace GC Ultra GC (Thermo Fisher Scientific) connected to a Delta^plus^ XL IRMS (Thermo Fisher Scientific) according to the method described by Miyajima* et al.* (1995). The ^13^C/^12^C of CH_4_ in natural gas was measured with a Flash EA1112 elemental analyzer (Thermo Fisher Scientific) connected to a Delta V Advantage ConFlo IV system (Thermo Fisher Scientific) (Miyajima *et al.*, 1995). All isotope ratios are reported relative to international standards: Vienna Standard Mean Ocean Water for δD and δ^18^O, and Vienna Pee Dee Belemnite for δ^13^C. The standard deviations of δD and δ^18^O in groundwater and δ^13^C of DIC and CH_4_ were ±0.5‰, ±0.1‰, ±1‰, and ±0.3‰, respectively.

### Microbial cell counting and next-generation sequencing (NGS) analysis of 16S rRNA genes

Groundwater samples for microscopic observations were filtered using polycarbonate membrane filters (pore size, 0.2 μm; diameter, 25 mm) (Millipore). Total microbial cells trapped on the filters were stained with SYBR Green I (Thermo Fisher Scientific) (Yanagawa *et al.*, 2016). A LIVE/DEAD BacLight bacterial viability kit (Thermo Fisher Scientific) was used to obtain the ratio of live to total microbial cells (Vezzulli *et al.*, 2015). Stained cells were observed under a BX51 epifluorescence microscope equipped with a U-MNIB3 fluorescence filter (Olympus), and more than 20 microscopic fields (approximately 20 cells in each field) were counted for each sample. Cells were counted within 48 h of groundwater sampling.

To analyze the archaeal and bacterial communities in the groundwater, 10 L of groundwater samples was aseptically filtered using Sterivex-GV filter units (pore size, 0.22 μm) (Millipore). Bulk DNA was extracted from trapped cells using an ISOIL for Beads Beating kit (Nippon Gene). The V3–V4 region of the 16S rRNA gene was amplified using a primer set, Pro341F and Pro805R for prokaryotes (Takahashi *et al.*, 2014). PCR products were purified through a MultiScreen PCR_96_ filter plate (Millipore) and analyzed using an Agilent DNA 1000 kit on an Agilent 2100 Bioanalyzer system (Agilent Technologies) to detect primer dimers and obtain the average molecular weight of each product. The Illumina sequencing library was generated according to the method described by Takahashi *et al.* (2014). Sequencing was conducted using a 600-cycle paired-end MiSeq reagent kit v3 (Illumina) on an Illumina MiSeq platform. After sequencing was complete, an image analysis, base calling, and error estimations were performed using Illumina Real-Time Analysis software version 1.17.28. Bioinformatic processing was performed using a combination of Quantitative Insights Into Microbial Ecology (QIIME) version 1.9.1 and USEARCH version 6.1 (Caporaso *et al.*, 2010; Edgar, 2010). Only reads that had quality value scores of ≥20 for more than 98% of the sequence were extracted for further analyses. Potential chimeras were removed using USEARCH version 6.1. Quality-filtered reads were assigned to operational taxonomic units (OTUs) at a 97% similarity level, and the Good’s coverage, Chao 1, and Shannon indices were then calculated with QIIME version 1.9.1. Taxonomy was assigned using the Ribosomal Database Project (RDP) MultiClassifier version 2.11 with a confidence level of 80% (Wang *et al.*, 2007). Sequences were deposited under GenBank/ENA/DDBJ accession numbers DRA004443 and DRA005244.

### Measurement of potential microbial CH_4_ production

Thirty milliliters of each groundwater sample was anoxically injected into an autoclaved 70-mL serum bottle that was then tightly sealed with a sterile butyl-rubber stopper and aluminum crimp. To assess the potential for CH_4_ production by methanogenic archaea, groundwater samples were amended with acetate (20‍ ‍mM), methanol (20‍ ‍mM), formate (20‍ ‍mM), or H_2_/CO_2_ (80:20‍ ‍[v/v]; 150 kPa). Except for H_2_/CO_2_-amended bottles, the headspaces of serum bottles were filled with pure N_2_ at 150 kPa. These cultures were anoxically incubated without shaking at temperatures that were 10°C higher than those of the groundwater samples because the actual temperature in a deep aquifer is generally considered to be higher than that of a groundwater sample measured at the outflow of the well.

To assess the potential for CH_4_ production by a syntrophic microbial community of H_2_-producing fermentative bacteria and H_2_-utilizing methanogenic archaea, groundwater samples were amended with organic nutrients. The chemical components of organic matter contained in the deep aquifers of the Shimanto Belt currently remain unclear. Therefore, we used a mixture of yeast extract, peptone, and glucose (YPG) as organic substrates because this mixture is widely used for anaerobic cultures of microorganisms and may promote the rapid growth of microbial communities in groundwater. Groundwater samples were amended with 3 mL of YPG medium (10 g yeast extract, 10 g peptone, and 2 g glucose L^–1^ distilled water). As a killed control, groundwater samples were autoclaved and then supplemented with 3 mL of YPG medium. The headspaces of serum bottles were filled with pure N_2_ at 150 kPa. These cultures were anoxically incubated without shaking at the temperatures of the groundwater samples measured at the outflows of the wells and temperatures that were 10°C and 20°C higher than those measured at the outflows. As a control, cultures using groundwater samples without any amendment were also incubated.

All cultures were performed in triplicate. H_2_, N_2_, CH_4_, and CO_2_ concentrations in the headspaces were measured on a GC-2014 GC equipped with a TCD (Shimadzu) as described above.

Microorganisms that grew in cultures were identified according to the 16S rRNA gene clone library method. Briefly, cells in cultures were collected by centrifugation and lysed by lysozyme and proteinase K. Bulk DNAs were extracted using both phenol/chloroform/isoamyl alcohol and chloroform/isoamyl alcohol and purified with ethanol precipitation. Archaeal and bacterial 16S rRNA gene fragments were amplified by PCR from bulk DNA using the *Archaea*-specific primer set, 109aF and 915aR (Stahl and Amann, 1991; Großkopf *et al.*, 1998), and *Bacteria*-specific primer set, 8bF and 1512uR (Eder *et al.*, 1999). PCR products of the archaeal and bacterial 16S rRNA genes were cloned with a Zero Blunt TOPO PCR cloning kit (Invitrogen). The sequences of the inserted PCR products selected from recombinant colonies were elucidated with an Applied Biosystems 3730xl DNA analyzer (Life Technologies). A 3% distance level between sequences was considered to be the cut-off for distinguishing distinct OTUs. We identified the nearest relative of each OTU using the BLAST program (Altschul *et al.*, 1990). Neighbor-joining phylogenetic trees were constructed using the CLUSTAL X version 2.1 program (Larkin *et al.*, 2007). Bootstrap values were obtained from 1,000 replications and indicated at the branching points. Sequences were deposited under GenBank/ENA/DDBJ accession numbers LC123696 to LC123721 and LC194992 to LC194998.

## Results

### Physicochemical signatures of groundwater and natural gas

Anoxic groundwater and natural gas samples were collected from deep aquifers through six deep wells. The groundwater temperature measured at the outflows of the wells ranged between 24.9 and 50.0°C (Table 1). The pH of groundwater ranged between 7.1 and 7.4. The ORP was measured as an indicator of anoxic conditions in the deep aquifers and ranged between –231 and –141 mV. The EC, an indicator of salinity, ranged between 488 and 5,000‍ ‍mS‍ ‍m^–1^. These parameters were measured several times between May 2014 and May 2019, and no temporal variations were detected. The concentration ranges of various chemical components were measured, including Br^–^ and I^–^ (0.04 to 1.38‍ ‍mM and 0.05 to 0.95‍ ‍mM, respectively; both were particularly high in samples from GRY), HCO_3_^–^ (1.5 to 46‍ ‍mM), and DOC (0.05 to 0.83‍ ‍mM) (Table S2). PO_4_^3–^, NO_3_^–^, SO_4_^2–^, S^2–^, acetate, and formate were found at trace amounts or were below detection limits.

CH_4_ was the predominant component of natural gas (Table 1). The other principal components were CO_2_ and N_2_. In addition to CH_4_, natural gas collected from KGO contained a particularly large amount of CO_2_ (34.8‍ ‍vol.%). C_2_H_6_ and C_3_H_8_ were found at trace amounts. The hydrocarbon gas composition C_1_/(C_2_+C_3_) of natural gas samples ranged between 571 and 11,211. H_2_, O_2_, and C_4_H_10_ concentrations were found at trace amounts or were below detection limits.

### Stable isotopic signatures of groundwater and natural gas

**Fig. 2.**
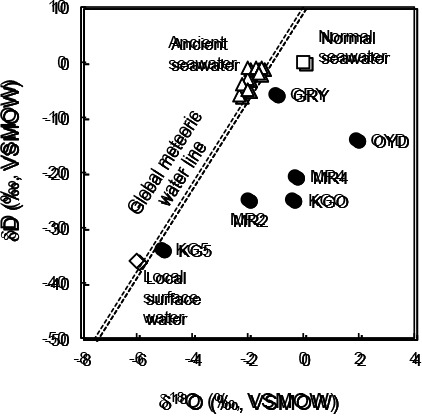
Stable hydrogen and oxygen isotope composition of groundwater samples (●) compared with those of local surface water (◇), normal seawater (□), and ancient seawater (△) (Maekawa *et al.*, 2006; Kato *et al.*, 2011). Dashed line: global meteoric water line (Craig, 1961). VSMOW, Vienna Standard Mean Ocean Water.

The δD and δ^18^O of groundwater samples ranged between –33.9 and –5.8‰ and between –5.1 and 1.9‰, respectively (Fig. 2 and Table S3). To estimate the origins of groundwater in the deep aquifers, we plotted δD and δ^18^O values with respect to the global meteoric water line (Craig, 1961). Groundwater sampled from KG5 was plotted closer to the local surface water (Kato *et al.*, 2011). The groundwater from OYD, MR2, MR4, and KGO fell in the upper right region of the local surface water. The groundwater from GRY was plotted closer to normal seawater and ancient seawater, as reported previously by Maekawa *et al.* (2006).

**Fig. 3.**
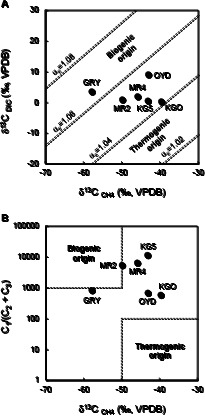
(A) Stable carbon isotope composition of CH_4_ in natural gas samples and dissolved inorganic carbon in groundwater samples. The categorization of CH_4_ origins was made according to Smith and Pallasser (1996). Dashed lines: equal carbon isotopic fractionation, α_c_=(δ^13^C_DIC_+10^3^)/(δ^13^C_CH__4_+10^3^), for α_c_=1.02, 1.04, 1.06, and 1.08. (B) Stable carbon isotope composition of CH_4_ and hydrocarbon gas composition C_1_/(C_2_+C_3_) in natural gas samples. The categorization of CH_4_ origins was made according to Bernard *et al.* (1978). VPDB, Vienna Pee Dee Belemnite.

The δ^13^C of DIC (consisting mainly of HCO_3_^–^) in groundwater (δ^13^C_DIC_) ranged between 0.16 and 8.95‰ (Fig. 3A and Table S3). The δ^13^C of CH_4_ in natural gas (δ^13^C_CH__4_) ranged between –57.8 and –39.6‰. To estimate the origin of CH_4_ in natural gas, we assessed carbon isotope fractionation (α_c_) between δ^13^C_DIC_ and δ^13^C_CH__4_. α_c_ values were 1.041 to 1.065 (Fig. 3A and Table S3). In a plot of stable isotopic values on a δ^13^C_DIC_ versus δ^13^C_CH__4_ diagram (Smith and Pallasser, 1996), the sample from GRY fell within the region of biogenic origin (α_c_=1.06–1.08). In contrast, all other sample values fell within the boundary between biogenic and thermogenic origins (α_c_=1.04–1.06), indicating that natural gas samples contained CH_4_ of mixed biogenic and thermogenic origins. We also plotted the observed isotopic values in a δ^13^C_CH__4_ versus hydrocarbon gas composition C_1_/(C_2_+C_3_) diagram according to Bernard *et al.* (1978). In this diagram, all sample values fell within the boundary between biogenic and thermogenic origins (Fig. 3B).

### Abundance and diversity of microbial communities in groundwater

The abundance of total microbial cells stained with SYBR Green I ranged between 8.2×10^4^ and 1.0×10^7^‍ ‍cells‍ ‍mL^–1^ (Table 2). Live/dead staining revealed that the density of live microbial cells ranged between 1.4×10^4^ and 1.4×10^6^‍ ‍cells‍ ‍mL^–1^ (Fig. S1). The percent cell viability (% live microbial cells/total microbial cells) ranged between 7.5 and 38.9% (Table 2).

To elucidate microbial community structures in anoxic groundwater derived from deep aquifers, we performed a NGS analysis targeting the archaeal and bacterial 16S rRNA genes. We obtained 12,678 to 54,714 reads and 234 to 804 OTUs (Table S4). Coverage reached >98.3%. The Chao1 and Shannon indices were 245 to 1,542 and 4.74 to 6.84, respectively.

**Fig. 4.**
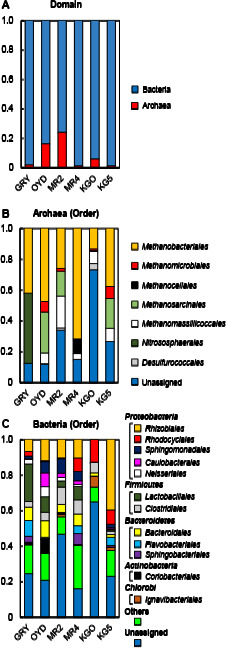
(A) Relative abundance of archaeal and bacterial 16S rRNA genes obtained from a NGS analysis. Taxonomic composition of microbial communities in natural groundwater samples based on the relative abundance of (B) archaeal and (C) bacterial 16S rRNA gene sequences. ‘Others’ indicate bacterial orders with relative abundance below 5% in all samples.

Archaeal 16S rRNA genes, which accounted for between 1.0 and 23.9% of the total reads obtained from each sample (Fig. 4A), revealed the predominance of methanogenic archaea belonging to the order *Methanobacteriales* (Fig. 4B). We also detected minor amounts of *Methanomicrobiales*, *Methanocellales*, and *Methanomassiliicoccales*. These methanogenic archaea are generally known to use H_2_ and CO_2_ to produce CH_4_, while some are capable of methylotrophic methanogenesis (Sakai *et al.*, 2008; Zhu *et al.*, 2011; Dridi *et al.*, 2012; Sakai *et al.*, 2012). In groundwater samples from OYD, MR2, and KG5, we also identified archaeal 16S rRNA genes that are closely related to those of *Methanosarcinales*, an order of methanogenic archaea that uses acetate, methanol, or H_2_ and CO_2_ as a methanogenic substrate (Kamagata *et al.*, 1992).

The analysis of bacterial 16S rRNA genes revealed the presence of bacterial groups that belong to the phyla *Proteobacteria*, *Firmicutes*, *Bacteroidetes*, *Actinobacteria*, and *Chlorobi* (Fig. 4C). At the order level, 6–40% of bacterial 16S rRNA genes obtained from all sites, except KGO, were closely related to *Rhizobiales*, an order of *Alphaproteobacteria*. The bacterial 16S rRNA genes closely related to the orders *Lactobacillales*, *Clostridiales*, and *Bacteroidales* were also detected from all sites. The bacterial groups of the orders *Coriobacteriales* and *Ignavibacteriales* were identified mainly in OYD and KGO, respectively.

### Potential for microbial CH_4_ production

To assess the potential for CH_4_ production by methanogenic archaea in the deep aquifers, we incubated groundwater samples amended with methanogenic substrates (acetate, methanol, formate, or H_2_/CO_2_) under anaerobic conditions. However, CH_4_ production was not observed in these cultures after incubations of longer than 60 d.

**Fig. 5.**
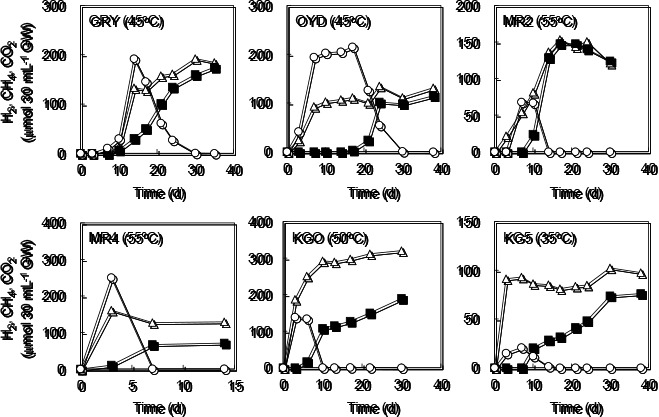
Biogas production from groundwater samples amended with YPG medium. The gas phase composition over time is shown as follows: H_2_ (○); CH_4_ (■); and CO_2_ (△). Incubation temperatures are shown in parentheses. Although representative results of cultures performed in triplicate are shown, all cultures showed similar potential for biogas production.

On the other hand, the high potential for CH_4_ production by microbial communities was confirmed in cultures using YPG medium-amended groundwater (Fig. 5 and S2). In cultures using groundwater from OYD, MR4, KGO, and KG5, the production of H_2_ and CO_2_ was observed within 3 d. In cultures using groundwater from GRY and MR2, H_2_ and CO_2_ were detected after 7 d. After the production of H_2_ and CO_2_, the concentration of H_2_ decreased to below the detection limit in all cultures. CH_4_ production was observed after H_2_ levels began to fall. In cultures using groundwater from GRY, MR2, MR4, KGO, and KG5, CH_4_ production was observed within 10 d. In cultures using groundwater from OYD, CH_4_ was detected after 17 d. In a killed control using the groundwater samples amended with YPG medium, abiotic H_2_ and CH_4_ production was not observed during an incubation of more than 120 d. Similarly, H_2_ and CH_4_ production was not observed in cultures using groundwater without organic substrates.

To identify microorganisms that generated biogases (*i.e.*, H_2_, CO_2_, and CH_4_), we constructed archaeal and bacterial 16S rRNA gene clone libraries. The 16S rRNA gene analysis suggested that H_2_-utilizing methanogenic archaea and H_2_-producing fermentative bacteria were predominant in the‍ ‍microbial population (Table S5) and were related to the‍ ‍archaeal orders *Methanobacteriales* and *Methanomicrobiales* (Fig. S3) and the bacterial orders *Bacillales*, *Bacteroidales*, *Clostridiales*, *Tissierellales*, and *Ignavibacteriales*, respectively (Fig. S4) (Parshina *et al.*, 2003; Watanapokasin *et al.*, 2009). Additionally, bacterial 16S rRNA genes closely related to the order *Desulfovibrionales*, which is known to form syntrophic microbial communities with H_2_-utilizing methanogens, were also confirmed in the clone libraries associated with GRY and OYD (Morris *et al.*, 2013).

## Discussion

To characterize the subsurface environments and identify the potential for CH_4_ production in anoxic deep aquifers associated with the Shimanto Belt and its overlying sedimentary layers in the southeastern part of Kyushu Island, Japan, we herein revealed the geochemical and microbiological features of deep aquifer-derived anoxic groundwater and natural gas samples. Groundwater from the KG5 site had the lowest EC value (488‍ ‍mS‍ ‍m^–1^), which was approximately 10% that of normal seawater (Table 1), and had similar δD and δ^18^O values to those of the local surface water (Fig. 2). Collectively, these characteristics suggest that the KG5 deep aquifer was affected by rainwater that flowed down from surface environments. In contrast, groundwater from GRY had almost the same EC value as normal seawater and ancient seawater (Table 1). The δD and δ^18^O values of groundwater sampled from the GRY site were also plotted closer to those of normal seawater and ancient seawater (Fig. 2). Additionally, the groundwater from GRY contained higher levels of Br^–^ and I^–^ than normal seawater (Table S2) (Millero *et al.*, 2008). These chemical features are consistent with those of ancient seawater, which is comprised of groundwater that originated from seawater and was maintained for a long period in a low-temperature deep aquifer (Katayama *et al.*, 2015). Therefore, the GRY deep aquifer was likely influenced by ancient seawater. This result differs from that previously reported in deep aquifers found in other regions of the Shimanto Belt that are affected by rainwater and normal seawater from surface environments (Matsushita *et al.*, 2016; Matsushita *et al.*, 2018).

The EC values of groundwater from OYD, MR2, MR4, and KGO were between approximately 20 and 44% that of normal seawater, suggesting that the deep aquifers at these sites hold groundwater derived from a mixture of rainwater and seawater that flowed down from the surface and the seafloor, respectively (Table 1). However, the δD and δ^18^O values of OYD, MR2, MR4, and KGO fell to the right of a line joining the plots of surface water and seawater, suggesting large δ^18^O enrichment (Fig. 2). The high δ^18^O values suggest that the groundwater in these deep aquifers was affected by water-rock interactions in thermal subsurface environments (Bowers and Taylor, 1985). Alternatively, these isotopic characteristics may be the result of groundwater evaporation due to geothermal heat (Connolly *et al.*, 1990).

CH_4_ was the predominant component of all natural gas samples with the exception of KGO, which also contained particularly high amounts of CO_2_ (34.8‍ ‍vol.%) and CH_4_ (62.9‍ ‍vol.%) (Table 1). Kato *et al.* (2011) reported that CO_2_ in natural gas from the KGO site is of magmatic origin based on its δ^13^C value. The accumulation of magmatic CO_2_ in natural gas has frequently been found in tectonically active oil- and gas-bearing basins (*e.g.*, Dai *et al.*, 1999). The highest groundwater temperatures recorded in the present study were also from the KGO site (50°C). Thus, the deep aquifer at the KGO site may be affected by magmatic CO_2_ and geothermal groundwater that rose from deep subsurface environments of active volcanoes, *e.g.*, the Kirishima Volcano and Sakurajima Volcano (Fig. 1). These results are distinct from previous findings on the deep aquifers of the Shimanto Belt, which is found in the mid-western part of Shizuoka Prefecture and the southern part of Okinawa Island, Japan (Matsushita *et al.*, 2016; Matsushita *et al.*, 2018).

The results of chemical and isotopic analyses of groundwater and natural gas samples suggested that the environmental features of the deep aquifers markedly differed among the sites studied herein. In contrast, stable carbon isotope analyses and microbiological assays suggested the presence of CH_4_ production by a syntrophic community of H_2_-producing fermentative bacteria and H_2_-utilizing methanogenic archaea in the deep aquifers. The δ^13^C_CH__4_ versus δ^13^C_DIC_ diagram and δ^13^C_CH__4_ versus C_1_/(C_2_+C_3_) diagram both indicated that all natural gas samples contained CH_4_ of biogenic origin or a mixture of CH_4_ of biogenic and thermogenic origins (Fig. 3). The NGS analysis of target archaeal 16S rRNA genes revealed the predominance of H_2_-utilizing methanogenic archaea belonging to the orders‍ ‍*Methanobacteriales*, *Methanomicrobiales*, and *Methanomassiliicoccales* in the groundwater samples (Fig. 4B). The NGS analysis of target bacterial 16S rRNA genes‍ ‍indicated the presence of bacteria belonging to the orders *Lactobacillales*, *Clostridiales*, *Bacteroidales*, *Coriobacteriales*, and *Ignavibacteriales* (Fig. 4C). These bacterial groups have the ability to degrade organic matter to H_2_ and CO_2_ by fermentation (Benno *et al.*, 1983; Kandler *et al.*, 1983; Liu *et al.*, 2008; Podosokorskaya *et al.*, 2013). The presence of *Rhizobiales* in groundwater samples was also confirmed. Although *Rhizobiales* is generally known to comprise aerobic diazotrophs, some species that belong to this order may grow by fermentation and are present in deep subsurface environments (Kodama and Watanabe, 2011; Miettinen *et al.*, 2015; Dutta *et al.*, 2018). The presence of methanogenic archaea and fermentative bacteria identified in the present study has also been reported in deep aquifers of other regions associated with the Shimanto Belt (Matsushita *et al.*, 2016; Matsushita *et al.*, 2018). Additionally, these microorganisms have frequently been found in subsurface gas and oil reservoirs, coal deposits, and subseafloor sediment cores in which microbial hydrogenotrophic CH_4_ production has been confirmed (Colwell *et al.*, 2004; Meslé *et al.*, 2013; Katayama *et al.*, 2015; Hirai *et al.*, 2017). These results suggest that a syntrophic community of H_2_-producing fermentative bacteria and H_2_-utilizing methanogenic archaea contributes to CH_4_ production in the deep aquifers tested. The microbial CH_4_ production process may be flexible to environmental differences by changing the microbial community structure while maintaining similar metabolic functions.

The microbial cell densities observed in the present study were similar to or higher than those previously reported in deep aquifers of the accretionary prism and other geothermal aquifers (Table 2) (Katayama *et al.*, 2015; Matsushita *et al.*, 2016; Matsushita *et al.*, 2018). These results also suggest the presence of microbial activity in the deep aquifers. Additionally, this is the first study to measure live cell density as well as total cell density in the groundwater of the Shimanto Belt. Groundwater from GRY showed relatively high live cell density (Table 2). Since CH_4_ from GRY was suggested to be of biogenic origin by the δ^13^C_DIC_ versus δ^13^C_CH__4_ diagram (Fig. 3A), the impact of high concentrations of Br^–^ and I^–^ on microbial CH_4_ production appears to be small. In contrast, the lowest live cell density was observed in KGO. This may be due to magmatic CO_2_ and geothermal groundwater from deep subsurface environments. The factors that influence microbial viability in deep aquifers may be more complex, such as the residence time of groundwater and the degradation rate of dead cells.

Although the potential for CH_4_ production was not confirmed in cultures using H_2_/CO_2_-amended groundwater, this may have been due to the growth inhibition of H_2_-utilizing methanogenic archaea caused by high concentrations of H_2_ and CO_2_ (Sakai *et al.*, 2009). On the other hand, the addition of organic substrates to groundwater samples demonstrated a high potential for H_2_ and CO_2_ production by H_2_-producing fermentative bacteria as well as rapid H_2_ consumption and significant CH_4_ production by H_2_-utilizing methanogenic archaea (Fig. 5 and S2). Since we used high concentrations of organic substrates, the microbial activity observed in the cultures does not reflect the activity level in the actual subsurface environment. Changes in microbial community structures between natural groundwater and culture samples, particularly bacterial communities, are attributed to the strong selective pressure caused by using YPG medium (Fig. 4 and Table S5). On the other hand, the present results showed high live cell densities and a high potential for microbial CH_4_ production. Thus, previous and ongoing microbial CH_4_ production appears to contribute to the significant CH_4_ reserves in the deep aquifers. A previous study reported that the average contents of total organic carbon in the sedimentary layers of the Nichinan Group and Miyazaki Group were 0.86 and 0.58%, respectively (Maki *et al.*, 1980). These contents are consistent with those of total organic carbon in the sediments of other accretionary prisms containing biogenic CH_4_ (Davie and Buffett, 2003; Oba *et al.*, 2015). Therefore, this organic matter appears to support the activity of microbial communities that generate CH_4_ in the deep aquifers. Further clarification of the composition of this organic matter is of fundamental importance for future studies.

The NGS analysis of archaeal 16S rRNA genes in natural groundwater samples revealed the presence of *Methanosarcinales*, which generally uses acetate or methanol for CH_4_ production (Fig. 4B). Additionally, *Methanobacteriales* and *Methanomassiliicoccales* identified in the present study grow by methylotrophic methanogenesis. On the other hand, CH_4_ production was not confirmed in cultures using groundwater amended with acetate, methanol, and formate. These results suggest that the potential for CH_4_ production using acetate, methanol, and formate in the deep aquifers is lower than that of H_2_-utilizing methanogenesis. This is consistent with the low concentrations of these methanogenic substrates in the groundwater (Table S2). However, we cannot exclude the possibility that CH_4_ production from these methanogenic pathways occurs in the deep aquifers because the culture conditions used in the present study were significantly different from the actual subsurface environments and may not have been suitable for these methanogenic archaea.

## Conclusion

In the present study, we demonstrated the potential for CH_4_ production by a syntrophic microbial community of H_2_-producing fermentative bacteria and H_2_-utilizing methanogens in deep aquifers that have widely varying geological and geochemical features. The production process of CH_4_ is similar to that previously reported in deep aquifers found in other regions of the Shimanto Belt that are affected by rainwater and normal seawater from surface environments (Matsushita *et al.*, 2016; Matsushita *et al.*, 2018). Since accretionary prisms are derived from ancient marine sediments scraped from the subducting ocean plate, they are rich in complex organic matter (Berner and Koch, 1993; Kaneko *et al.*, 2010). The results of the present study support a model in which the biodegradation of organic matter in ancient sediments contributes to the generation of CH_4_ reserves in deep aquifers associated with accretionary prisms and their overlying sedimentary layers, which are distributed globally and have diverse geological and geochemical features.

## Supplementary Material

Supplementary Material

## Figures and Tables

**Table 1.** Physical and chemical parameters of groundwater and components of natural gas.

Site code	Well depth (Strainer depth) (m)	Groundwater					Natural gas			
		Temp. (ºC)	pH	ORP^a^ (mV)	EC^b^ (mS m^–1^)		N_2_ (vol.%)	CO_2_ (vol.%)	CH_4_ (vol.%)	C_1_/(C_2_+C_3_)^c^
GRY	1,000 (591–983)	24.9	7.1	–231	5,000		0.6	<0.01	99.2	813
OYD	1,301 (947–1,240)	44.5	7.4	–197	2,220		3.0	2.90	94.0	666
MR2	847 (711–831)	38.1	7.4	–181	1,064		3.2	1.75	95.0	5,280
MR4	1,054 (774–1,054)	44.7	7.3	–169	1,409		3.2	2.10	94.7	6,312
KGO	810 (581–779)	50.0	7.2	–199	1,009		2.2	34.80	62.9	571
KG5	1,070 (925–1,047)	35.1	7.2	–141	488		3.5	6.80	89.7	11,211

^a^ ORP, oxidation-reduction potential. ^b^ EC, electrical conductivity. ^c^ C_1_, CH_4_; C_2_, C_2_H_6_; C_3_, C_3_H_8_.

**Table 2.** Microbial cell density and cell viability in groundwater.

Site code	Total cell density (cells mL^–1^)	Live cell density (cells mL^–1^)	Cell viability^a^ (%)
GRY	1.0×10^7^	1.3×10^6^	13.0
OYD	5.1×10^6^	3.8×10^5^	7.5
MR2	3.6×10^6^	1.4×10^6^	38.9
MR4	1.7×10^6^	5.2×10^5^	30.6
KGO	8.2×10^4^	1.4×10^4^	17.1
KG5	5.2×10^5^	1.2×10^5^	23.1

^a^ Cell viability=(% live cells/total cells).
