# Supplementary material for: The Potential for CH4 Production by Syntrophic Microbial Communities in Diverse Deep Aquifers Associated with an Accretionary Prism and its Overlying Sedimentary Layers
Source: Microbes Environ. 2020 Jan 11;35(1):ME19103. doi: 10.1264/jsme2.ME19103 (PMC7104277; doi:10.1264/jsme2.ME19103)
Supplement: Supplementary file 1 — Supplementary Material [file 35_19103_s1.pdf]

Table S1. Location and structure of deep wells used for sampling.

Sampling site	Site code	Latitude	Longitude	Well depth (Strainer depth)	Formation of deep aquifer
Gokuraku-yu	GRY	31°55'09.46"N	131°25'35.37"E	1,000 m (591-983 m)	Miyazaki Group
Oyodo-onsen	OYD	31°54'24.33"N	131°25'34.04"E	1,301 m (947-1,240 m)	Miyazaki Group
Mingas-R2	MR2	31°56'18.76"N	131°23'12.78"E	847 m (711-831 m)	Nichinan Group
Mingas-R4	MR4	31°55'55.79"N	131°24'08.41"E	1,054 m (774-1,054 m)	Nichinan Group
Kitago-R1	KGO	31°39'29.48"N	131°23'07.56"E	810 m (581-779 m)	Miyazaki Group and Nichinan Group
Kitago-R5	KG5	31°41'48.86"N	131°22'07.60"E	1,070 m (925-1,047 m)	Nichinan Group

Table S2. The chemical characteristics of groundwater, surface seawater, and ancient seawater.

Site code	Na^+ (mM)	Ca^{2+} (mM)	Mg^{2+} (mM)	K^+ (mM)	NH_4^+ (mM)	Cl^- (mM)	Br^- (mM)	I^- (mM)	F^- (mM)	PO_4^{2-} (mM)	NO_3^- (mM)	SO_4^{2-} (mM)	S^{2-} (mM)	HCO_3^- (mM)	Acetate (mM)	Formate (mM)	DOC[a] (mM)
GRY	435	10.0	6.6	1.1	2.3	451	1.38	0.95	0.050	<0.005	<0.02	<0.001	<0.02	1.5	<0.08	<0.1	0.83
OYD	170	2.7	1.3	0.7	0.5	152	0.21	0.24	0.080	<0.005	<0.02	0.001	<0.02	11.0	<0.08	<0.1	0.07
MR2	87	0.4	0.3	0.4	0.2	51	0.10	0.09	0.210	<0.005	<0.02	<0.001	<0.02	23.0	<0.08	<0.1	0.11
MR4	117	0.7	0.5	0.7	0.3	82	0.14	0.14	0.150	<0.005	<0.02	0.001	<0.02	21.0	<0.08	<0.1	0.07
KGO	109	1.1	0.4	1.3	0.7	54	0.09	0.09	0.090	<0.005	<0.02	0.002	<0.02	46.0	<0.08	<0.1	0.07
KG5	32	2.5	2.1	0.3	0.2	28	0.04	0.05	0.005	<0.005	<0.02	0.001	<0.02	13.0	<0.08	<0.1	0.05
Normal seawater[c]	469	10.0	53.0	10.0	NA[b]	546	0.84	0.0004	NA	NA	NA	28	NA	1.7	NA	NA	NA
Ancient seawater[d]	357	3.0	15.0	6.4	28	508	1.75	0.94	NA	0.03	<0.0001	<0.005	NA	18.0	<0.002	NA	NA

[a] DOC, dissolved organic carbon.

[b] NA, data not available.

[c] Taken from Millero *et al*. (2008) *Deep Sea Res., Part I* **55**: 50–72.

[d] Taken from Katayama *et al*. (2015) *ISME Journal* **9**: 436–446.

Table S3. Stable isotopic signatures of groundwater and natural gas.

Site code	Groundwater			Natural gas	α_c [a]
	δD of H_2O (‰, VSMOW[c])	$\delta^{18}O$ of H_2O (‰, VSMOW[c])	$\delta^{13}C_{DIC}$ [b] (‰, VPDB[d])	$\delta^{13}C_{CH4}$ (‰, VPDB[d])	
GRY	−5.8	−1.0	3.42	−57.8	1.065
OYD	−14.0	1.9	8.95	−43.0	1.054
MR2	−24.6	−2.0	0.78	−49.8	1.053
MR4	−20.7	−0.3	1.83	−45.8	1.050
KGO	−24.9	−0.4	0.16	−39.6	1.041
KG5	−33.9	−5.1	0.41	−43.1	1.045

[a] $\alpha_c = (\delta^{13}C_{DIC} + 10^3)/(\delta^{13}C_{CH4} + 10^3)$.

[b] DIC, dissolved inorganic carbon.

[c] VSMOW, Vienna Standard Mean Ocean Water.

[d] VPDB, Vienna Pee Dee Belemnite.

Table S4. Number of prokaryotic 16S rRNA gene sequences derived from groundwater and statistical estimators.

Site code	Total of sequences	No. of OTUs[a]	Coverage (%)	Chao 1	Shannon
GRY	12,678	387	99.5	441	5.78
OYD	18,703	444	99.0	563	6.35
MR2	16,263	487	99.0	606	6.84
MR4	14,931	234	99.8	245	6.51
KGO	54,714	804	99.3	1,542	4.74
KG5	19,166	609	98.3	822	6.43

[a] OTUs, operational taxonomic units.

Table S5. Archaeal and bacterial 16S rRNA gene sequences derived from the cultures using groundwater amended with YPG medium.

Site code	OTU[a]	Accession no.	No. of clones	Phylogenetic group	Closest cultivated species (% identity)	Predicted metabolism[b]
GRY	*Archaea*					
	GRY_cA01	LC123715	52	*Methanobacteriales*	*Methanobacterium aarhusense* (99)	HM[c]
	Total		52			
	Bacteria					
	GRY_cB01	LC123716	54	*Bacillales*	*Paenibacillus polymyxa* (83)	FE[d]
	GRY_cB02	LC123717	2	*Desulfovibrionales*	*Desulfovibrio alkalitolerans* (99)	FE
	GRY_cB03	LC123718	2	*Clostridiales*	*Caldicoprobacter oshimai* (86)	FE
	Total		58			
OYD	*Archaea*					
	OYD_cA01	LC123705	49	*Methanobacteriales*	*Methanobacterium beijingense* (96)	HM
	OYD_cA02	LC123706	3	*Methanobacteriales*	*Methanobacterium aarhusense* (99)	HM
	Total		52			
	Bacteria					
	OYD_cB01	LC123707	40	*Bacteroidales*	*Proteiniphilum acetatigenes* (99)	FE
	OYD_cB02	LC123708	8	*Ignavibacteriales*	*Melioribacter roseus* (99)	FE
	OYD_cB03	LC123709	7	*Flavobacteriales*	*Schleiferia thermophila* (84)	AHT[e]
	OYD_cB04	LC123710	4	*Bacteroidales*	*Acetobacteroides hydrogenigenes* (86)	FE
	OYD_cB05	LC123711	2	*Desulfovibrionales*	*Desulfovibrio alkalitolerans* (100)	FE or SR[f]
	OYD_cB06	LC123712	1	*Sphingomonadales*	*Sphingopyxis alaskensis* (100)	AHT
	OYD_cB07	LC123713	1	*Rhodocyclales*	*Thauera aminoaromatica* (95)	FE
	OYD_cB08	LC123714	1	*Flavobacteriales*	*Flavobacterium psychrophilum* (96)	AHT
	Total		64			
MR2	*Archaea*					
	MR2_cA01	LC123701	51	*Methanobacteriales*	*Methanobacterium aarhusense* (99)	HM
	Total		51			
	Bacteria					
	MR2_cB01	LC123702	36	*Clostridiales*	*Caldicoprobacter oshimai* (83)	FE
	MR2_cB02	LC123703	10	*Spirochaetales*	*Treponema stenostreptum* (88)	FE
	MR2_cB03	LC123704	5	*Bacteroidales*	*Mariniphaga anearophila* (90)	FE
	Total		51			
MR4	*Archaea*					
	MR4_cA01	LC123696	48	*Methanobacteriales*	*Methanothermobacter thermautotrophicus* (99)	HM
	Total		48			
	Bacteria					
	MR4_cB01	LC123697	47	*Bacteroidales*	*Acetobacteroides hydrogenigenes* (86)	FE
	MR4_cB02	LC123698	7	*Anaerolineales*	*Bellilinea caldifistulae* (99)	FE
	MR4_cB03	LC123699	3	*Coriobacteriales*	*Eggerthella lenta* (89)	FE
	MR4_cB04	LC123700	1	*Tissierellales*	*Soehngenia saccharolytica* (99)	FE
	Total		58			
KGO	*Archaea*					
	KGO_cA01	LC194992	67	*Methanobacteriales*	*Methanothermobacter thermautotrophicus* (100)	HM
	Total		67			
	Bacteria					
	KGO_cB01	LC194993	59	*Tissierellales*	*Soehngenia saccharolytica* (99)	FE
	KGO_cB02	LC194994	10	*Tissierellales*	*Soehngenia saccharolytica* (99)	FE
	KGO_cB03	LC194995	8	*Thermoanaerobacterales*	*Tepidanaerobacter syntrophicus* (93)	FE
	KGO_cB04	LC194996	1	*Thermoanaerobacterales*	*Tepidanaerobacter syntrophicus* (93)	FE
	KGO_cB05	LC194997	1	*Tissierellales*	*Soehngenia saccharolytica* (99)	FE
	KGO_cB06	LC194998	1	*Tissierellales*	*Sporanaerobacter acetigenes* (87)	FE
	Total		80			
KG5	*Archaea*					
	KG5_cA01	LC123719	50	*Methanomicrobiales*	*Methanolinea tarda* (95)	HM
	Total		50			
	Bacteria					
	KG5_cB01	LC123720	33	*Ignavibacteriales*	*Melioribacter roseus* (91)	FE
	KG5_cB02	LC123721	17	*Spirochaetales*	*Treponema stenostreptum* (88)	FE
	Total		50			

[a] OTU, operational taxnomic unit.

[b] Predicted metabolism was referred to that of each type strain.

[c] HM, H_2-utilizing methanogenesis.

[d] FE, fermentation.

[e] AHT, aerobic heterotroph.

[f] SR, sulfate reduction.

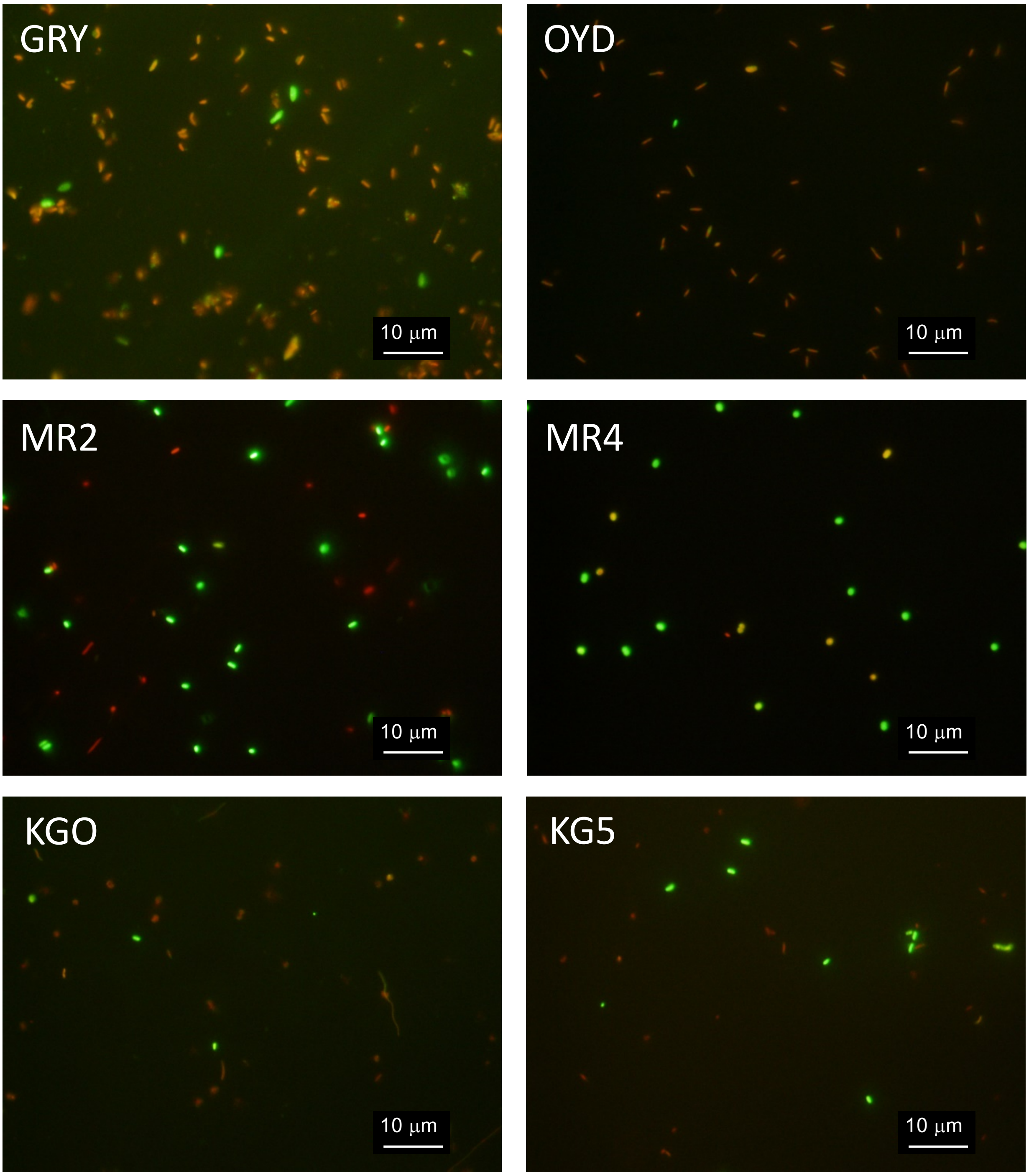

Fig. S1. Epifluorescence micrographs of LIVE/DEAD staining microbial cells in groundwater samples. Green signals represent live microbial cells, which have an intact cell membrane. Yellow and red signals are dead microbial cells, which have a damaged cell membrane.

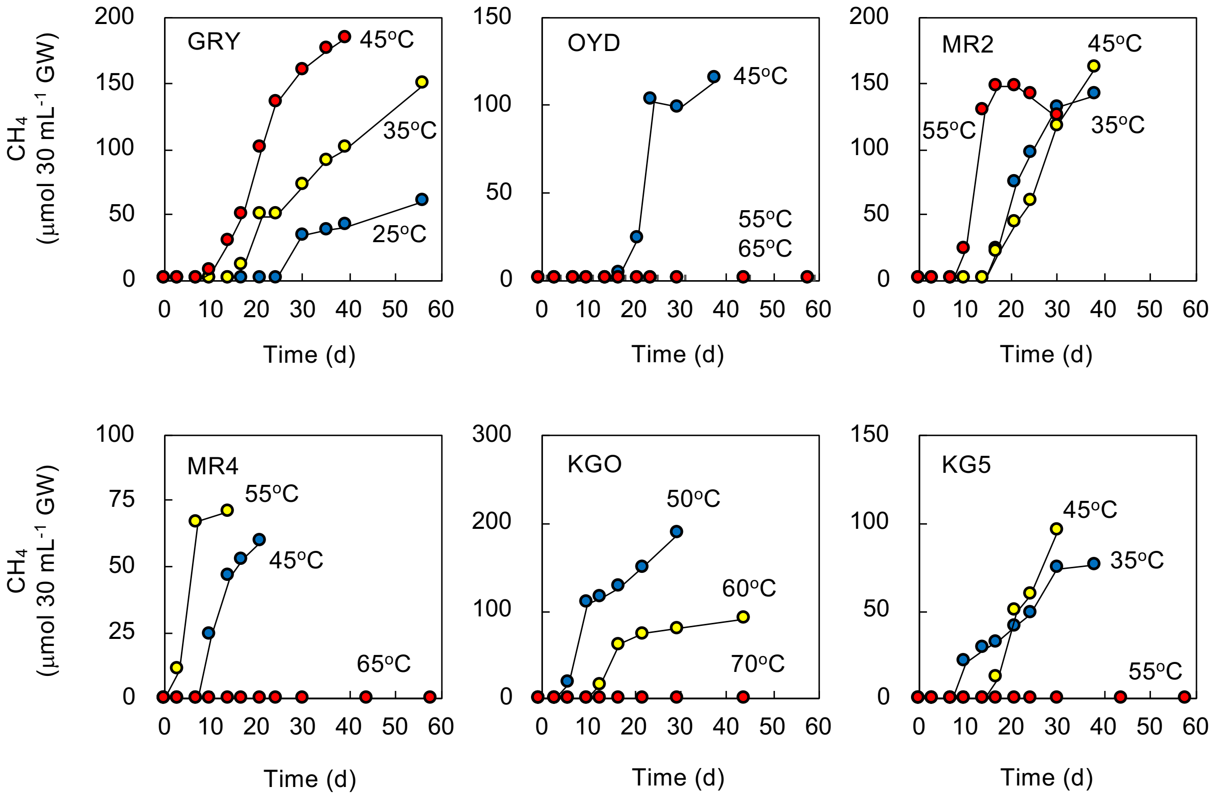

Fig. S2. CH_4 production from groundwater samples amended with YPG medium. Data points were obtained from the measurement of cumulative CH_4 in gas phase of bottled cultures. Cultures were anaerobically incubated without shaking at temperatures of the groundwater samples measured at the outflow of the wells (blue circles) and temperatures that were 10°C (yellow circles) and 20°C (red circles) higher than those measured at the outflow of the wells. Although representative results of cultures performed in triplicate are shown, all cultures showed similar potential for biogas production.

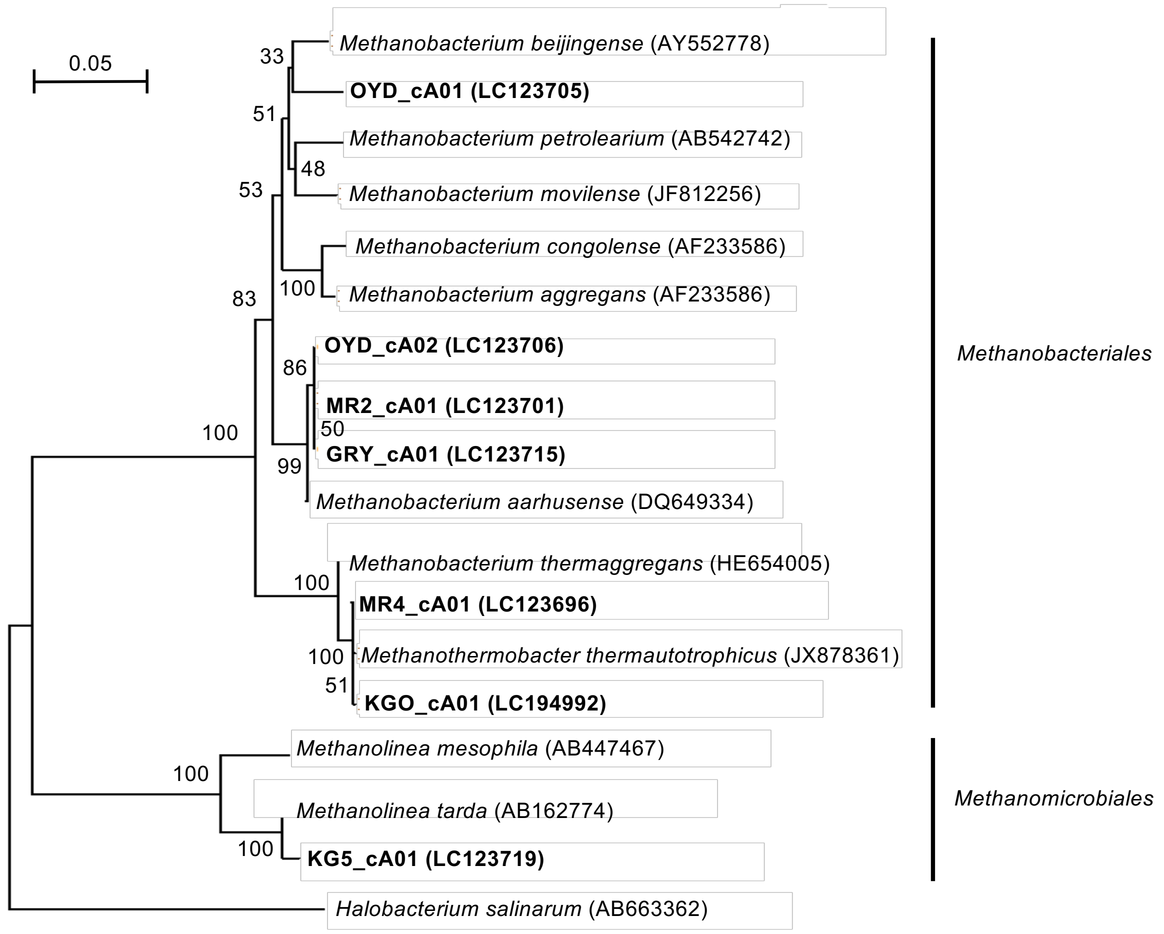

Fig. S3. Neighbor-joining tree of the archaeal 16S rRNA gene sequences derived from the cultures using YPG medium-amended groundwater for CH_4 production. 7 OTUs obtained in this study are shown with their relatives of established species whose sequences were retrieved from the public database. Accession numbers are shown in parentheses. Alignment length was 807 bp. Bootstrap values determined from 1,000 iterations are indicated at branching points. The sequence of *Halobacterium salinarum* was used as the outgroup to root the tree. Scale bar represents 5 substitution per 100 nucleotides.

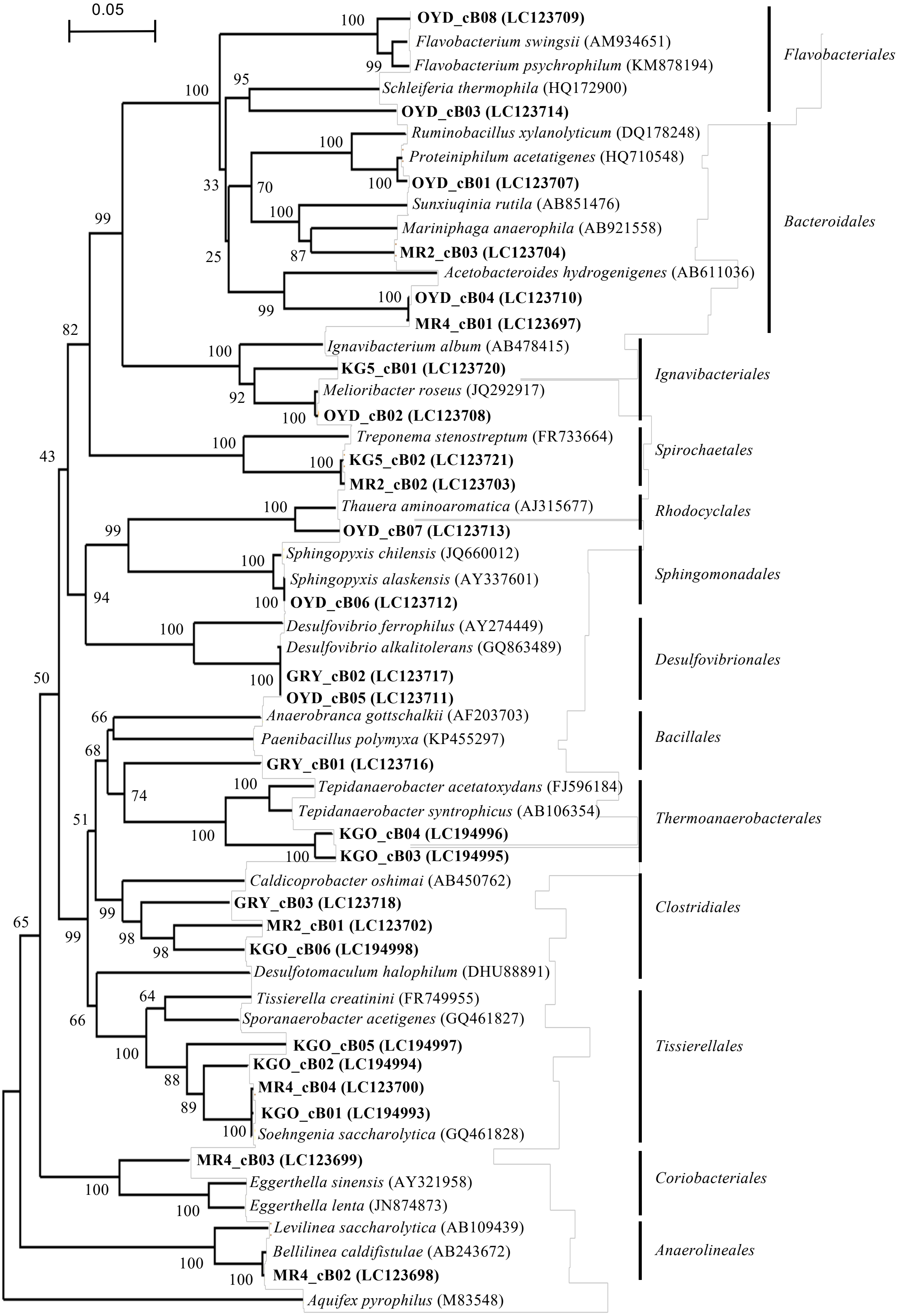

Fig. S4. Neighbor-joining tree of the bacterial 16S rRNA gene sequences derived from the cultures using YPG medium-amended groundwater for CH_4 production. 26 OTUs obtained in this study are shown with their relatives of established species whose sequences were retrieved from the public database. Accession numbers are shown in parentheses. Alignment length was 1505 bp. Bootstrap values determined from 1,000 iterations are indicated at branching points. The sequence of *Aquifex pyrophilus* was used as the outgroup to root the tree. Scale bar represents 5 substitution per 100 nucleotides.
